# Carbon Fibers Embedded With Iron Selenide (Fe_*3*_Se_*4*_) as Anode for High-Performance Sodium and Potassium Ion Batteries

**DOI:** 10.3389/fchem.2020.00408

**Published:** 2020-06-03

**Authors:** Asif Mahmood, Zeeshan Ali, Hassina Tabassum, Aftab Akram, Waseem Aftab, Rashad Ali, Muhammad Waqas Khan, Suraj Loomba, Ahmed Alluqmani, Muhammad Adil Riaz, Muhammad Yousaf, Nasir Mahmood

**Affiliations:** ^1^School of Chemical and Biomolecular Engineering, The University of Sydney, Sydney, NSW, Australia; ^2^School of Chemical and Materials Engineering, National University of Sciences and Technology, Islamabad, Pakistan; ^3^Beijing Key Laboratory for Theory and Technology of Advanced Battery Materials, Department of Material Science and Engineering, College of Engineering, Peking University, Beijing, China; ^4^School of Materials and Energy, University of Electronic Science and Technology of China, Chengdu, China; ^5^School of Engineering, RMIT University, Melbourne, VIC, Australia; ^6^Applied Porous Materials Unit, Commonwealth Scientific and Industrial Research Organisation (CSIRO), Clayton, VIC, Australia; ^7^International Center for Quantum Materials and Electron Microscopy Laboratory, School of Physics, Peking University, Beijing, China

**Keywords:** iron selenide, sodium ion batteries, potassium ion batteries, anodes, hierarchical structures

## Abstract

The development of sodium and potassium ion batteries (SIBs/KIBs) has seen tremendous growth in recent years due to their promising properties as a potential replacement for lithium-ion batteries (LIBs). Here, we report ultrafine iron selenide (Fe_3_Se_4_) nanoparticles embedded into one-dimensional (1D) carbon fibers (Fe_3_Se_4_@CFs) as a potential candidate for SIBs/KIBs. The Fe-based metal-organic framework particles (MOFP) are used as a Fe source to obtain highly dispersed Fe_3_Se_4_ nanoparticles in the product. The Fe_3_Se_4_@CF consisted of ultrafine particles of Fe_3_Se_4_ with an average particle size of ~10 nm loaded into CFs with an average diameter of 300 nm. The product exhibited excellent specific activity of ~439 and ~435 mAh/g at the current density of 50 mA/g for SIBs and KIBs, respectively. In addition, the as-prepared anodes (Fe_3_Se_4_@CFs) exhibited excellent capacity retention up to several hundred cycles (700 cycles for SIBs and 300 cycles for KIBs). The high activity and excellent stability of the developed electrodes make Fe_3_Se_4_@CFs a promising electrode for next-generation batteries.

## Introduction

The electrochemical energy storage devices have found wide-scale applications from portable hand-held devices to grid-scale energy storage (Yu et al., 2014; Xia et al., 2015; Jiang et al., 2016a; Su et al., 2017). Among electrochemical energy storage devices, the lithium-ion batteries (LIBs) have found commercial-scale applications; however, the limited Li reserves and high cost severely limit the long-term reliance on lithium battery technology (Mahmood and Hou, 2014; Cha et al., 2017; Yang et al., 2018; Yousaf et al., 2018; Zhang et al., 2019). Recently, efforts have been made to replace the lithium metal with more abundant and cost-effective sodium and potassium metals to pave the way for sodium-ion batteries (SIBs) and potassium ion batteries (KIBs), respectively (Zou et al., 2017; Guo et al., 2018; Vaalma et al., 2018). The sodium and potassium are located in the same group of the periodic table as lithium, offer similar charge storage mechanisms, and are abundantly present in the Earth's crust all around the world. However, the application of sodium and potassium in battery technology is severely affected by limited insertion capacity of Na^+^/K^+^ ions in graphite (~35 mAh/g) due to their much larger ionic size in comparison to Li^+^ ions (Li et al., 2016; Tian et al., 2018; Jian et al., 2019; Mahmood et al., 2019). Therefore, intensive efforts are needed to develop high-performing new anode materials to expedite the development of SIBs/KIBs.

The transition metal-based electrodes have been proposed as potential candidates for high-performance anode materials. For example, Fe_2_O_3_, CoSe_2_, TiO_2_, Ti_3_C_2_, etc., have shown promising charge storage capability for SIBs/KIBs (Yao et al., 2016; Dong et al., 2017; Ren et al., 2017; Ali et al., 2018a, 2019; Zhou et al., 2020). Among transition metal compounds, the transition metal chalcogenides (TMCs) have been reported as a possible solution to promote the reversible capacity of the anode materials for SIBs/KIBs due to their robust nature, high theoretical capacity and low cost (Yousaf et al., 2019; Ali et al., 2020). Among several chalcogenides, iron selenides like FeSe_2_, FeSe, and Fe_7_Se_8_ have shown promising storage capacities, cycling stabilities, and rate capabilities (Jiang et al., 2016b; Park et al., 2016; Ge et al., 2018; Wan et al., 2018). However, like all other TMCs, iron selenides undergo large volume change upon reversible conversion reaction with Na^+^/K^+^, which may cause quick decay in cycling capacity. This volume change is more pronounced in KIBs owing to relatively larger K^+^ ions (Luo et al., 2015; Zou et al., 2017; Tabassum et al., 2019). Different strategies have been proposed to address these challenges such as confining active species in the conductive carbon matrix, downsizing the TMCs to nanoscale and creating porosity in the electrodes for fast mass diffusion, etc. (Ali et al., 2020). However, finding a most suitable strategy for these emerging battery technologies remains elusive.

Here, we present a facile methodology to develop iron selenide (Fe_3_Se_4_) embedded into one-dimensional (1D) carbon fibers decorated with iron selenide (Fe_3_Se_4_@CFs) as an anode for SIBs/KIBs. To the best of our knowledge, there is no report on the development and utilization of the Fe_3_Se_4_ phase of iron selenides family for SIBs/KIBs. The Fe_3_Se_4_ is a stable phase and possesses a sufficiently high theoretical capacity of 443.5 mAh/g^−1^. 1D morphology was chosen to obtain fast conductive pathways for electronic/ionic transport and accommodate the volume variation during insertion/de-insertion; the hierarchical structure formed as a result of fiber agglomerations. The hierarchical structure provided the necessary porosity for mass transport and increased electrode/electrolyte interface. The iron-based metal-organic framework particles (MOFP) were used to homogeneously disperse the metallic species in CFs (Mahmood et al., 2015, 2016). The Fe_3_Se_4_@CFs exhibited excellent reversible capacity of ~439 and ~435 mAh/g at the current density of 50 mA/g for SIBs and KIBs. We believe that the presented methodology will offer a unique way to tailor high-capacity electrodes for SIBs/KIBs.

## Experimental Section

### Synthesis of MOFPs

The Fe-based MOFPs were synthesized using our previously reported method (Mahmood et al., 2015, 2018). In a typical synthesis method, iron nitrate non-ahydrate (Fe(NO_3_)_3_.·9H_2_O, 10 mmol) and benzene tricarboxylate (H_3_BTC, 15 mmol) were mixed in ethanol to get the MOFPs. The mixtures were transferred to a Teflon-lined steel vessel for aging at 120°C for 24 h. The obtained products were washed with ethanol and dried for further processing.

### Synthesis of MOFP/PAN Fibers

The MOFPs (0.5 g) and polyacrylonitrile (PAN) (0.5 g) were dissolved in dimethylformamide (DMF) to form a viscous solution. The solution was stirred overnight at room temperature. The resulting solution was then subjected to electrospinning at a flow rate of 0.8 ml/h. The product was dried at 60°C for 12 h and collected for further processing.

### Synthesis of Fe@CF

The MOFP/PAN fibers were calcined at 800°C in Ar. The ramp rate was 5°C and the samples were kept in the furnace for 5 h.

### Synthesis of Fe_3_Se_4_@CFs

The Fe@CF was selenized at 650°C in Ar to obtain Fe_3_Se_4_@CFs. The Fe@CF was put in the crucible and covered with a thin layer of selenium. The weight ratio of Fe@C and Se was 1:5. The product was allowed to cool down to room temperature and was collected for further analysis.

### Characterization

The structural characterization was done by using field emission scanning electron microscope (FESEM, FEI Nova Nano SEM 430) and transmission electron microscope (TEM) and high-resolution TEM (HRTEM), FEI Tecnai T20). The X-ray diffraction (XRD) analysis was carried out by using Bruker D8 diffractometer.

### Electrochemical Characterizations

The electrochemical characterization was carried out using LAND 2001 analyzer. The sodium and potassium half-cells were assembled in CR2032 type coin cells. The active material was mixed with a conductive agent (carbon black) and polymer binder (carboxymethoxyl cellulose sodium (CMC)) in a ratio of 70:15:15 to develop the working electrode. The mixture was loaded onto the copper foil and dried at 80°C. The SIBs were assembled using a working electrode as an anode, sodium metal as counter electrode, and glass fiber paper as the separator. 1 M sodium tri-fluoro-methane-sulfonate (NaCF_3_SO_3_) dissolved in diglyme was used as an electrolyte for SIBs. For KIB, the potassium metal was used as a counter electrode and 1 M Potassium hexafluorophosphate (KPF_6_) dissolved in ethylene carbonate-diethylene carbonate (EC:DEC) as an electrolyte. The cyclic voltammetry (CV) and electrochemical impedance were tested using CHI workstation.

## Results and Discussions

**Figure 1:**
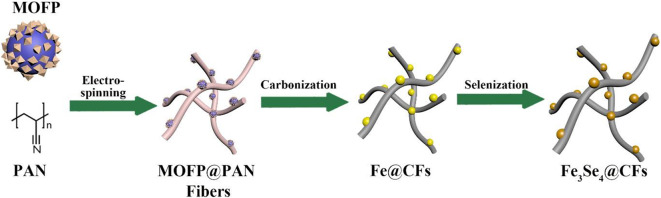
Schematic illustration of the growth process of Fe_3_Se_4_@CFs.

The 1D Fe_3_Se_4_@CFs were synthesized using a three-step methodology as presented schematically in Figure 1. The MOFPs, which we reported previously, were used as a source of Fe species in synthesis methodology (Mahmood et al., 2015, 2018, 2019). The MOFPs are particularly better at controlling metal distribution in the product and help in obtaining nanoscale control on the product. The MOFPs were dispersed in polyacrylonitrile (PAN) solution followed by electrospinning of the MOFP/PAN solution to obtain MOFP@PAN fibers. The MOFP/PAN was then carbonized at 800°C to obtain Fe@CF, followed by selenization in the presence of Se at 650°C in the tube furnace to finally achieve Fe_3_Se_4_@CFs.

**Figure 2:**
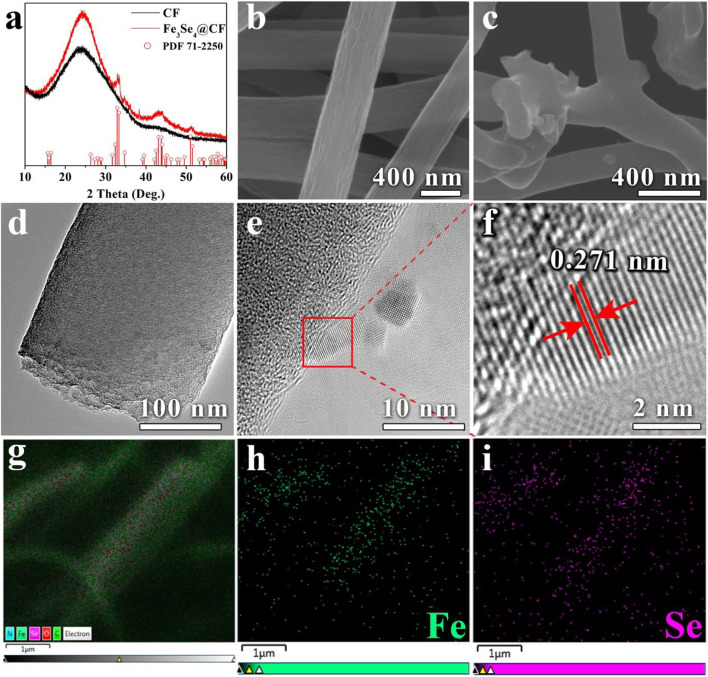
XRD analysis of CFs and Fe_3_Se_4_@CFs **(a)**, SEM analysis of Fe_3_Se_4_@CFs **(b,c)**, TEM and HRTEM analysis of Fe_3_Se_4_@CFs **(d,e)** EDS elemental mapping of Fe_3_Se_4_@CFs **(g–i)**.

The XRD analysis revealed the successful formation of Fe_3_Se_4_@CFs (Figure 2a). The diffraction pattern matched well with JCPDS card (71-2250) of Fe_3_Se_4_, which indicates the high purity of the product. A broad peak around 25° shows the presence of disordered carbon in the product. The FESEM analysis revealed a homogeneous distribution of carbon fibers with an average diameter of ~400 nm as shown in Figure 2b. The subsequent carbonization and selenization lead to a reduction in average diameter, with CFs in Fe_3_Se_4_@CFs exhibiting an average diameter of ~300 nm (Figure 2c). No metallic species were observed on the surface of the product, clearly indicating that the particles were embedded inside the framework of CFs. The TEM analysis was carried out to further investigate the microstructure of Fe_3_Se_4_@CFs and presented in Figures 2d,e. The low magnification TEM shows compact carbon fiber with interconnected porosity (Figure 2d) while high magnification confirmed the presence of Fe_3_Se_4_ on the fibers (Figure 2e). Furthermore, the HRTEM analysis revealed the existence of an interlayer spacing of 0.271 nm corresponding to the (112) plane of Fe_3_Se_4_, which matches with XRD analysis as shown in Figure 2f. Distribution of iron and selenium across the CFs was confirmed by employing EDS (energy-dispersive X-ray spectroscopy) technique and the results are presented in Figures 2g–i. It is apparent from EDS maps that the iron and selenium are uniformly distributed over CFs and no agglomerations of selenium were present.

**Figure 3:**
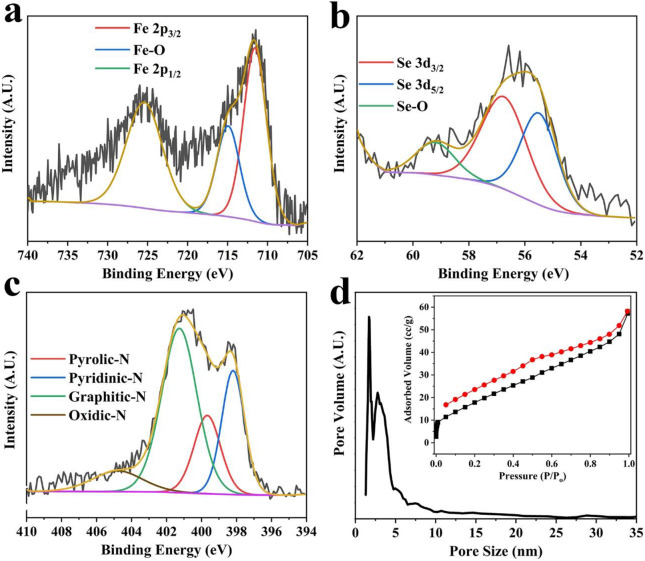
High-resolution XPS spectra of Fe **(a)**, Se **(b)**, and N **(c)** from Fe_3_Se_4_@CFs. The pore size distribution of Fe_3_Se_4_@CFs **(d)** while the inset shows the corresponding BET isotherm.

The elemental compositions and oxidation states of various constituents of Fe_3_Se_4_@CFs were estimated by X-ray photoelectron spectroscopy (XPS) analysis presented in Figures 3a–c and Figure S1. It is evident from these results that the Fe, Se, N, and C are mainly present in Fe_3_Se_4_@CFs samples, which indicates the high purity of the product. The XPS analysis revealed ~75% C, ~3.2 % Se, 4% N, and ~5.1% Fe in the product. In high resolution XPS, Fe 2p spectrum of Fe_3_Se_4_@CFs indicates two main peaks at around 711.8 and 725.9 eV, which corresponds to Fe 2p_3/2_ and Fe 2p_1/2_. One satellite peak centered around 716 eV is also observed assigned to Fe-O bond indicates oxidation states of Fe element (Figure 3a). On the other hand, the Se 3d spectra were best fitted with two peaks of Se 3d_3/2_ and Se 3d_5/2_ located around 55.4 and 54.2 eV, respectively (Figure 3b). The deconvolution of carbon 1s indicated the presence of C-C, C=C, and C-O, which correspond to the peaks at 285.2, 286.5, and 289.1 eV, respectively, as shown in Figure S1a in the Supporting Information. The Brunauer–Emmett–Teller (BET) isotherm shows a clear desorption hysteresis, indicating the presence of mesopores in the product. The Fe_3_Se_4_@CFs exhibited a surface area of 46.8 m^2^/g and predominantly pore size distribution in the mesoporous range (2–5 nm), which was calculated by using nonlinear density functional theory (NL-DFT) as shown in Figure 3d. The BET isotherm and pore size distribution results of bare CFs are presented in Figure S2 which show much larger pore sizes (> 10 nm) present in bare CFs. The existence of a relatively large number of pores in the Fe_3_Se_4_@CFs electrode ensures fast electrolyte diffusion of large Na/K-ions, leading to enhanced performances at higher charge/discharge rates.

**Figure 4:**
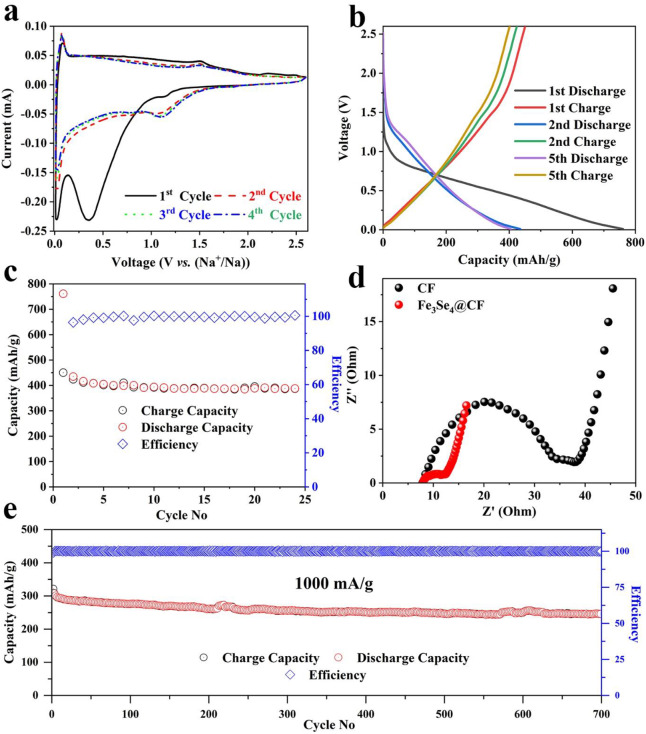
SIBs performance: CV analysis at scan the scan rate of 0.1 mV/s **(a)**, Galvanostatic charge/discharge profiles **(b)**, corresponding charge storage capacity at the current density of 50 mA/g **(c)**, comparison of EIS analysis **(d)**, cyclic life at a current density of 1,000 mA/g **(e)**.

The electrochemical properties of the developed electrode materials were tested for both SIBs and KIBs. The SIB half-cells were assembled using Fe_3_Se_4_@CFs as anode, sodium metal as a cathode, glass fiber paper as separator, and 1 M NaCF_3_SO_3_ dissolved in diglyme as the electrolyte. The cyclic voltammetry (CV) was used to investigate the sodium storage mechanism for the Fe_3_Se_4_@CFs in the range of 0.01 to 2.6 V as shown in Figure 4a. A clear cathodic peak was observed at 0.45 V in the first discharge cycle indicating irreversible reactions occurring on the surface of the electrode. The subsequent cycles exhibited two reversible oxidation peaks at 0.2 V and 1.6 V and a single reduction peak at 1.2 V. The appearance of peaks in subsequent cycles showed the reversible sodiation on the Fe_3_Se_4_@CFs. For comparison, CV analysis of a cell composed of bare CFs is presented in Figure S3. It is apparent that in all three cycles of bare CFs, typical peaks of Fe_3_Se_4_ are absent. The Galvanostatic charge-discharge curves (Figure 4b) further exhibited a plateau in the charge-discharge curves signifying the reversible redox reaction on the electrode surface. A plateau was observed close to 0.8 V in the first discharge curve followed by gradual sloping. The subsequent cycles exhibited short plateau at 1.4 V indicating the formation of Na_x_FeSe_2_, Na_2_Se and elemental iron (Zhao et al., 2017; Ali et al., 2018b)

In SIBs, the developed electrodes exhibited a capacity of 750 mAh/g for the first discharge while the subsequent cycles exhibited a reversible capacity of 439 mAh/g at a current density of 50 mA/g as shown in Figure 4c. The origin of higher capacity can be attributed to the highly active nature of Fe_3_Se_4_ toward sodium ions. The electrochemical impedance spectroscopic (EIS) analysis was carried out for bare and Fe_3_Se_4_@CF samples to further understand origin of high performance and gain deep insights into charge transfer kinetics of the SIBs (Yu et al., 2014). The EIS analysis revealed relatively higher internal resistance (R_CT_) value of 35 Ω for bare CFs. The Fe_3_Se_4_@CF, however, exhibited much lower R_CT_ of only 12 Ω as shown in Figure 4d. The EIS analysis was further extended to post electrochemical testing and the results are shown in Figures S4, S5. It is quite clear that the R_CT_ decreased to 8 ohms for Fe_3_Se_4_@CF based SIBs after testing which clearly indicate its excellent charge transfer kinetics (Figure S4). An increase in RCT was observed for CFs after cycling, which can be attributed to loss of electrical contact (Figure S5). In addition, the Fe_3_Se_4_@CFs exhibited excellent reversible capacity at a higher discharge rate of 1,000 mA/g for several hundred cycles as shown in Figure 4e. The Fe_3_Se_4_@CFs delivered about 80% capacity after 700 cycles with high coulombic efficiency near 100%. High capacity retention is ascribed to the highly flexible nature of CFs that acts as a cushion to buffer the volume expansion of active material (Fe_3_Se_4_) during the cycling process.

**Figure 5:**
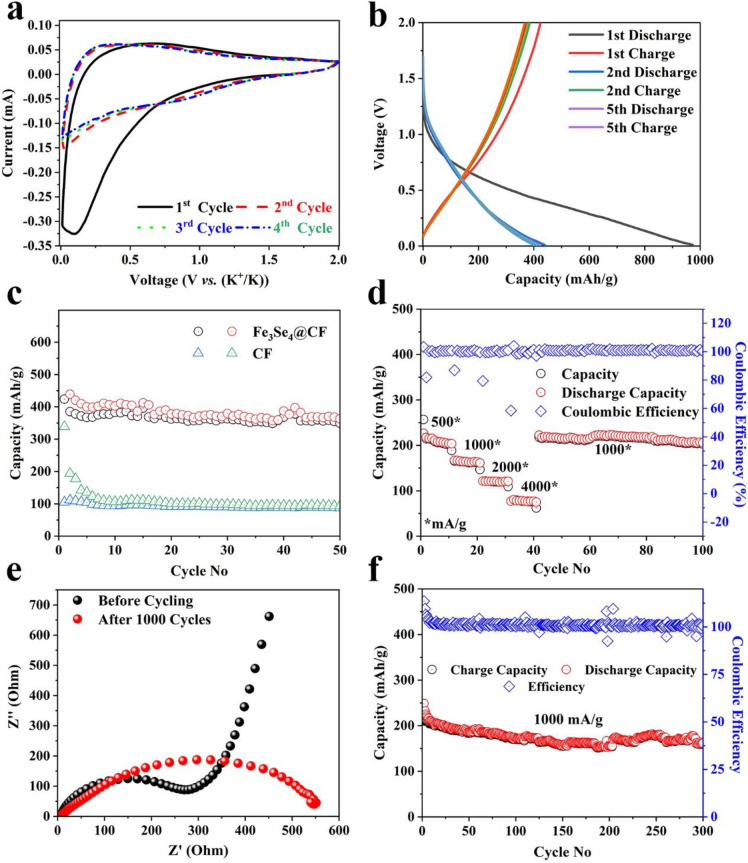
KIBs performance: CV analysis at a scan rate of 0.1 mV/s **(a)**, Galvanostatic charge/discharge profiles **(b)**, corresponding charge storage capacity at the current density of 50 mA/g **(c)**, charge storage capability at various current densities **(d)**, EIS analysis **(e)**, and cyclic life at a current density of 1,000 mA/g.

Inspired by excellent electrochemical performance for SIB, the Fe_3_Se_4_@CFs was further tested as anode material for KIBs. The KIB half-cells were assembled using Fe_3_Se_4_@CFs as an anode, potassium metal as a counter cathode, glass fiber paper as separator, and 1 M KPF_6_ dissolved in EC:DEC as an electrolyte. The CV analysis was carried out in a voltage window of 0.01-2 V and the results are shown in Figure 5a. The CV analysis revealed the origin of a reduction peak at about 1.25 V for several successive cycles. Furthermore, the Galvanostatic charge-discharge curves also indicate a plateau at ~1.1 V clearly indicating reversible phase transformation upon potassiation/de-potassiation as shown in Figure 5b. A specific capacity of ~960 mAh/g was observed for the first discharge at a current density of 50 mA/g. The subsequent cycles exhibited a fully reversible capacity of 435 mA/g for several cycles as shown in Figure 5c. In comparison to a much high specific capacity of Fe_3_Se_4_@CFs, the CFs exhibited a much lower specific capacity of 100 mAh/g which further demonstrates the high activity of Fe_3_Se_4_ toward K^+^ ion storage.

The transition metal electrodes generally fail to show good charge storage capability when tested under different current densities in KIBs, due to structural degradation of the electrodes. The Fe_3_Se_4_@CFs anode was tested at various current densities to investigate the rate capability and the results are shown in Figure 5d. The developed electrode exhibited excellent charge storage capacity of 203, 165, 120, and 77 mAh/g at corresponding current densities of 500, 1,000, 2,000, and 4,000 mA/g, respectively. The developed electrode exhibited an excellent capacity of 212 mAh/g upon returning to a current density of 1,000 mA/g. The excellent capacity can be attributed to much lower charge transfer resistance (R_CT_) of 290 Ω. The CFs, on the other hand, exhibited much higher R_CT_ of ~1,600 Ω (Figure S6), which indicates the higher charge transfer kinetics in Fe_3_S_4_ containing samples resulting in enhanced potassiation/de-potassiation (Figure 5e). The post-electrochemical EIS analysis suggested slightly increased resistance implying the electrolyte decomposition and slight loss in electrical contact as shown in Figure 5e. In addition to high rate capability, the Fe_3_Se_4_@CFs exhibited excellent capacity retention up to 300 cycles with a specific capacity of 161 mAh/g at a current density of 1000 mA/g (Figure 5f).

The high electrochemical storage of Na^+^ and K^+^ ions in Fe_3_Se_4_@CFs is due to many structural features. Firstly, the flexible and porous nature of CFs accommodates the strains produced during the electrochemical process, while its 1D nature provides straight paths for the transport of charges. Secondly, the macroporosity between the CFs allows the infiltration of electrolytes leads to shortened Na^+^ and K^+^ diffusion paths and enhanced the migration speed. Thirdly, the ultra-small and homogenously embedded Fe_3_Se_4_ NPs into CFs provide enormous active sites to accelerate Na^+^ or K^+^ insertion/extraction process.

## Conclusions

In summary, we have presented 1D carbon fibers containing highly active redox-active species (Fe_3_Se_4_) as anodes for next-generation battery technologies (SIBs/KIBs) beyond LIBs. The developed electrode consisted of ultrafine Fe_3_Se_4_ particles dispersed in the CF backbone and exhibited excellent charge storage capability for Na^+^/K^+^ ion storage. The Fe_3_Se_4_@CFs exhibited an excellent specific charge storage capacity of ~400 mAh/g for SIB/KIB at a current density of 50 mA/g. The higher capacity is attributed to the presence of highly dispersed ultrafine Fe_3_Se_4_ nanoparticles which offered reversible conversion reaction with the Na^+^/K^+^ ions. In addition, the electrode exhibited excellent cyclic stability for several hundred cycles further demonstrate that the developed electrode materials are highly reversible. We believe that the presented methodology will pave new ways in tailoring highly active anode chemistries for SIBs/KIBs.

## Data Availability Statement

The original contributions presented in the study are included in the article/Supplementary Materials, further inquiries can be directed to the corresponding author.

## Author Contributions

AM and NM conceptualized the work. AM, ZA, and HT synthesized the products. WA, RA, SL, and MY, did the electrochemical testing. AA helped in revising the manuscript. MK helped in experimentation requested during the review of the manuscript. All authors contributed to the article and approved the submitted version.

## Conflict of Interest

The authors declare that the research was conducted in the absence of any commercial or financial relationships that could be construed as a potential conflict of interest.
