# Supplementary material for: Carbon Fibers Embedded With Iron Selenide (Fe3Se4) as Anode for High-Performance Sodium and Potassium Ion Batteries
Source: Front Chem. 2020 Jun 3;8:408. doi: 10.3389/fchem.2020.00408 (PMC7283878; doi:10.3389/fchem.2020.00408)
Supplement: Supplementary file 1 [file Data_Sheet_1.docx]

**Supporting Information**

**Carbon Fibers Embedded with Iron Selenide (Fe_3_Se_4_) as Anode for High-Performance Sodium and Potassium Ion Batteries**

Asif Mahmood,^a^* Zeeshan Ali,^b^ Hassina Tabassum,^c^ Aftab Akram,^b^ Waseem Aftab,^c^ Rashad Ali,^d^ Muhammad Waqas Khan,^e,f^ Suraj Loomba,^e^ Ahmed Alluqmani,^e^ Muhammad Adil Riaz,^a^ Muhammad Yousaf,^c,g^ Nasir Mahmood,^e^*

^a^Dr. A. Mahmood, M. A. Riaz

School of Chemical and Biomolecular Engineering, The University of Sydney, Darlington, Sydney, New South Wales, Australia, 2006

^b^Dr. Z. Ali, Dr. A. Akram

School of Chemical and Materials Engineering, National University of Sciences and Technology, Islamabad 44000, Pakistan

^c^Dr. H. Tabassum, Dr. W. Aftab, Dr. M. Yousaf

Beijing Key Laboratory for Theory and Technology of Advanced Battery Materials, Department of Material Science and Engineering, College of Engineering, Peking University, Beijing, 100871, China

^d^R. Ali

School of Materials and Energy, University of Electronic Science and Technology of China, Chengdu, 611731, China

^e^M. W. Khan, S. Loomba, A. Alluqmani, Dr. N. Mahmood

School of Engineering, RMIT University, 124 La Trobe Street, 3001 Melbourne, Victoria, Australia

^f^M. W. Khan

Applied Porous Materials Unit, Commonwealth Scientific and Industrial Research Organisation (CSIRO), Clayton, VIC 3168, Australia

^g^Dr. M. Yousaf

International Center for Quantum Materials and Electron Microscopy Laboratory, School of Physics, Peking University, Beijing 100871, China

**Corresponding Authors:**

Dr. Asif Mahmood: [asif.mahmood@sydney.edu.au](mailto:asif.mahmood@sydney.edu.au),

Dr. Nasir Mahmood: [nasir.mahmood@rmit.edu.au](mailto:nasir.mahmood@rmit.edu.au)


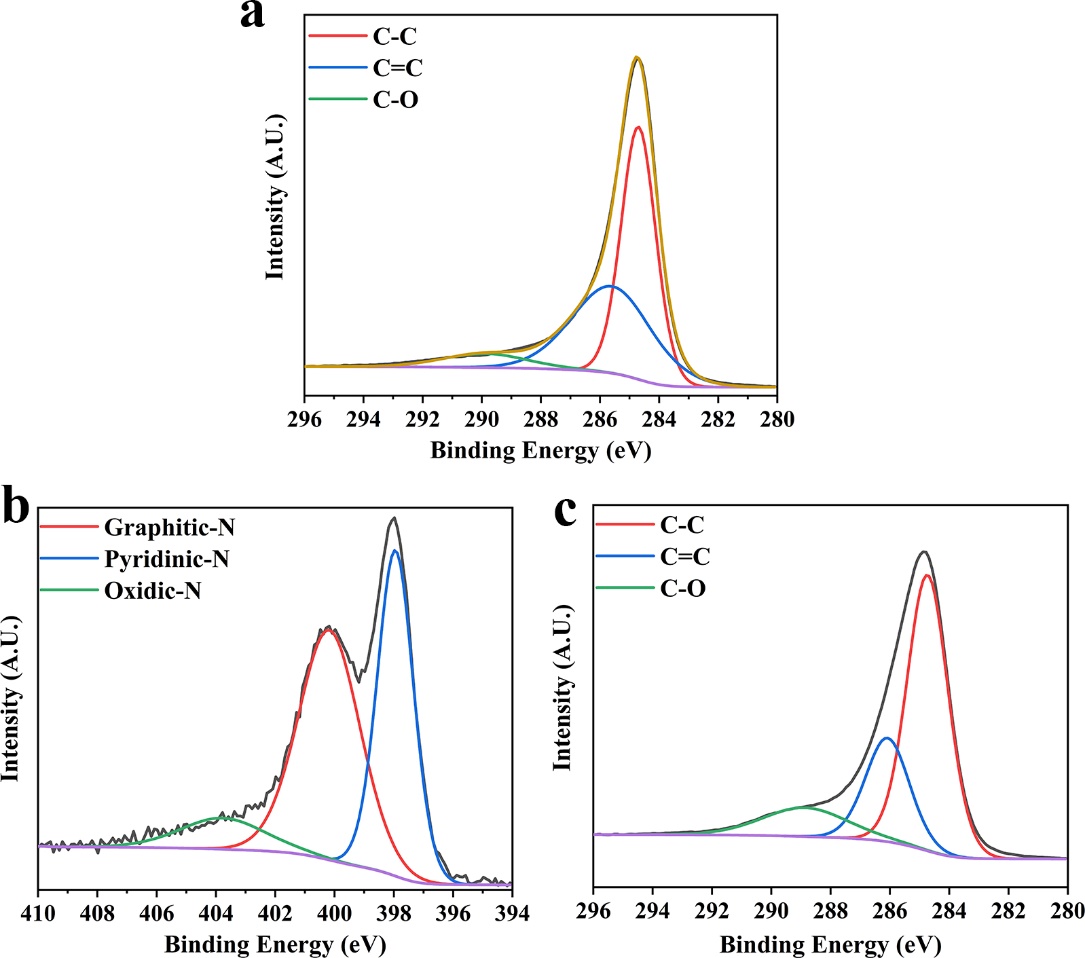


**Figure S1.** (a) Deconvolution of C from Fe_3_Se_4_@CF. XPS analysis of CFs: deconvoluted spectra of (b) N and (c) C


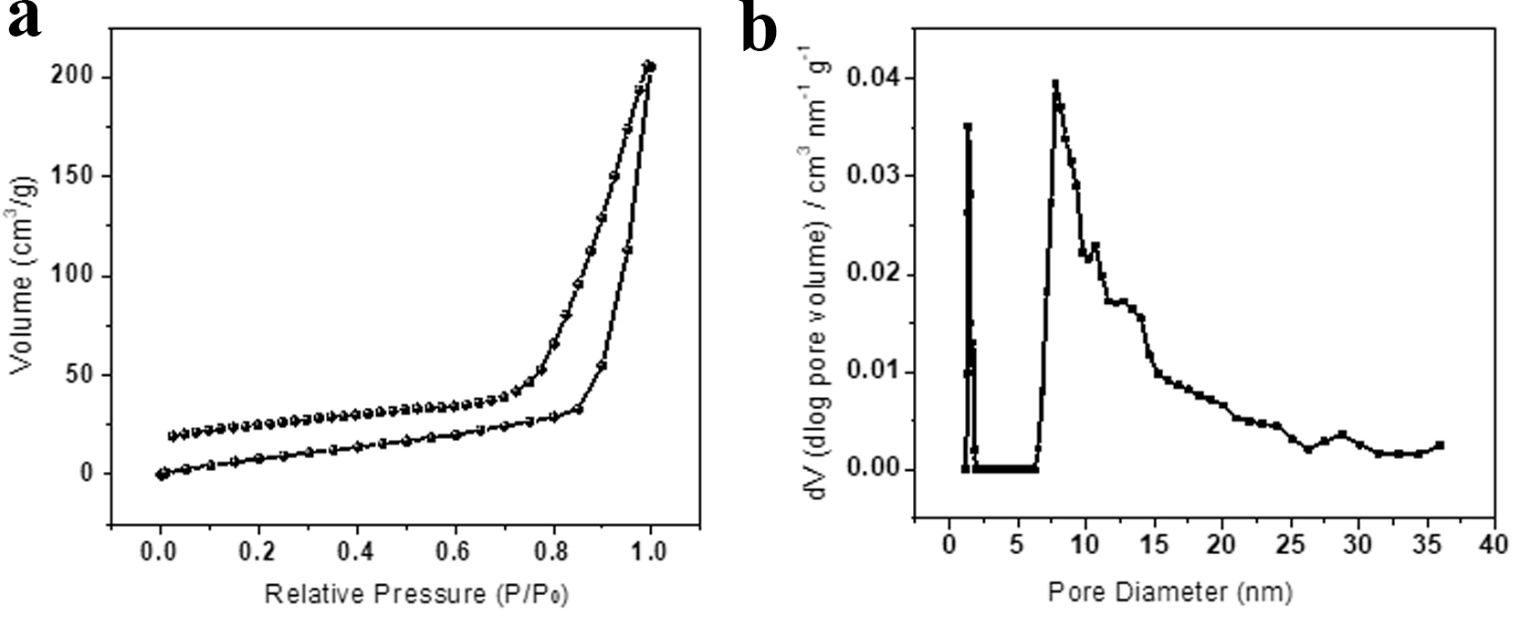


**Figure S2.** BET analysis of CF. (a) isotherm (b) pore size distribution


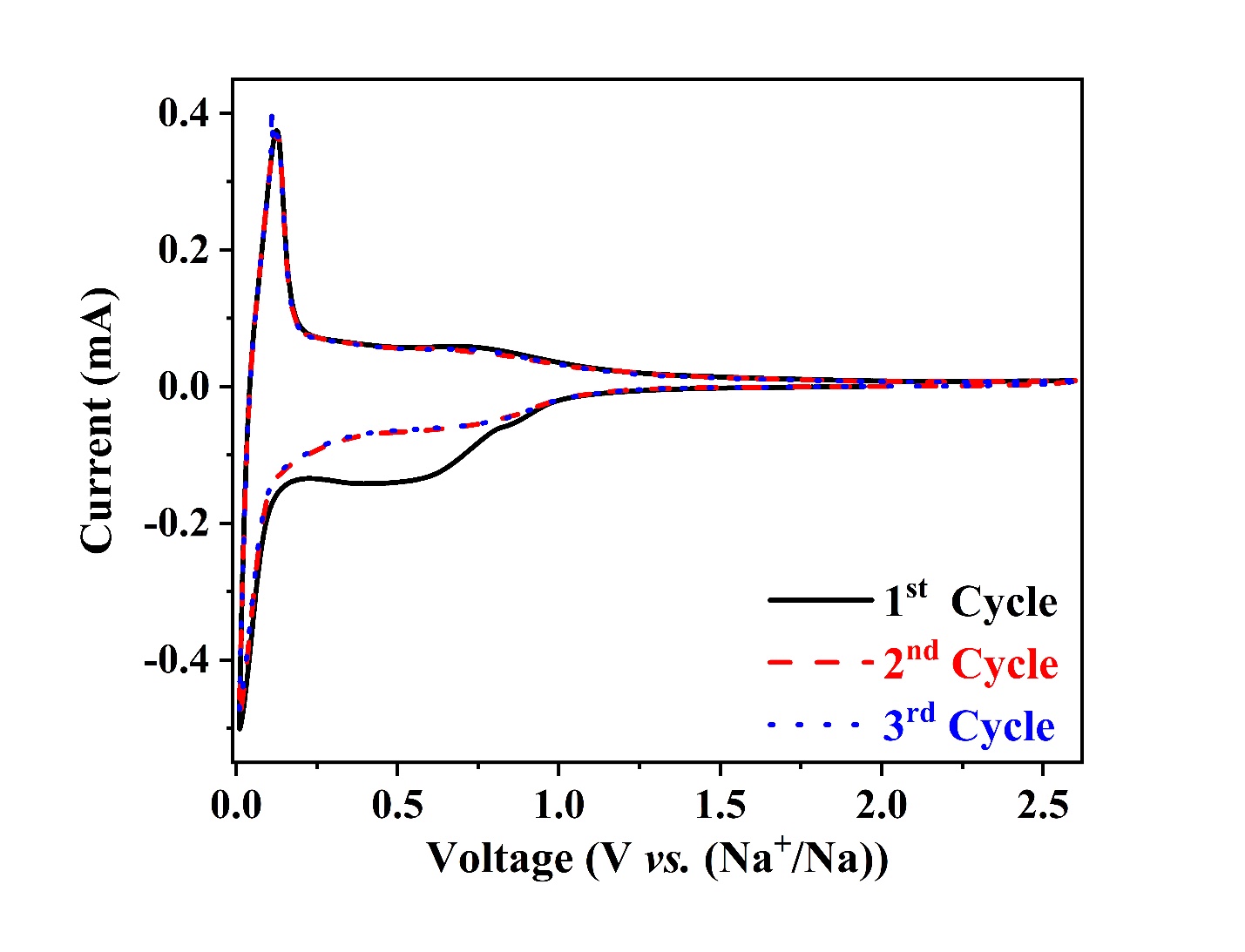


**Figure S3.** CV analysis of CF for sodium ion battery


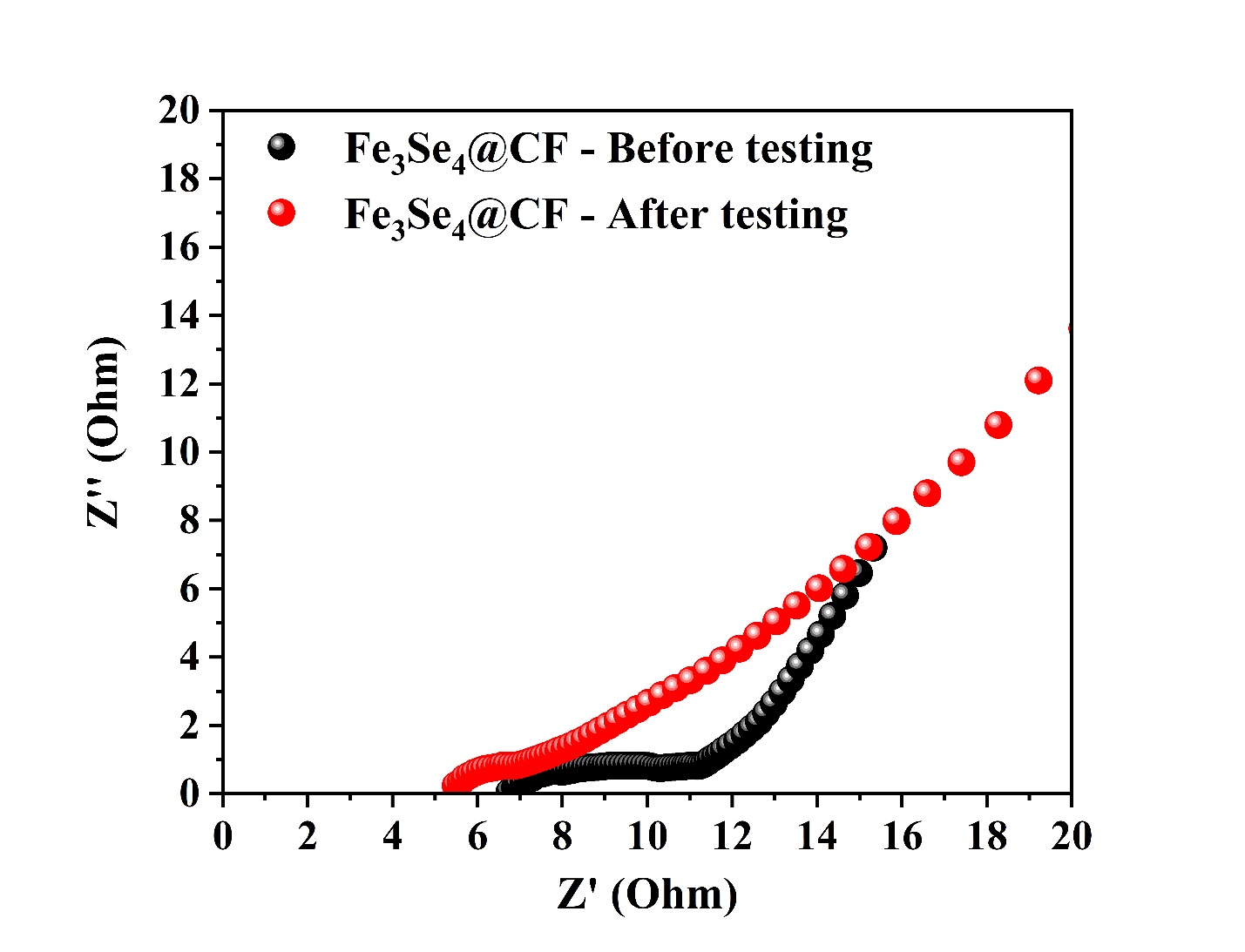


**Figure S4.** SIB analysis. EIS analysis of Fe_3_Se_4_@CF before and after CV analysis


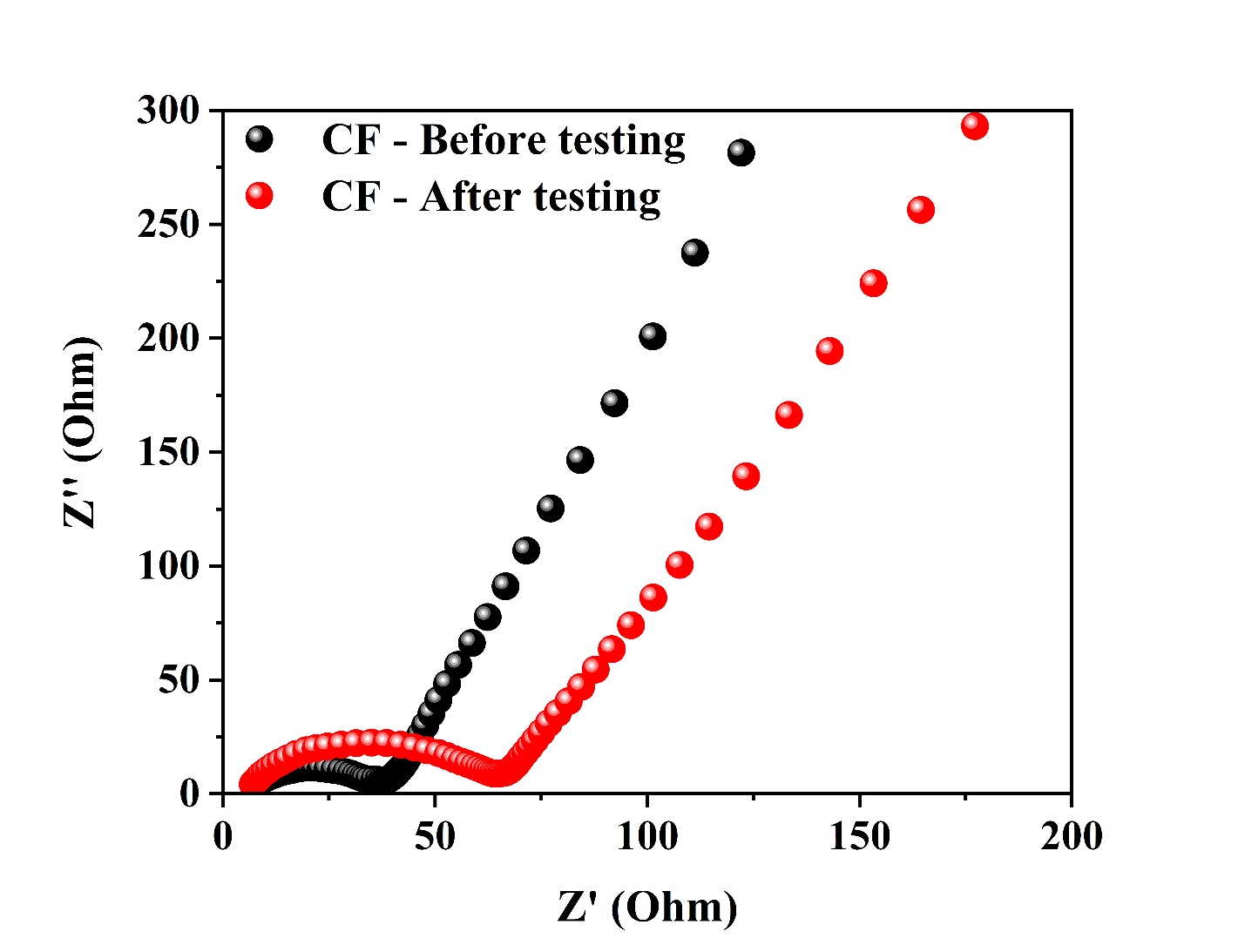


**Figure S5.** SIB analysis. EIS analysis of CFs before and after CV analysis


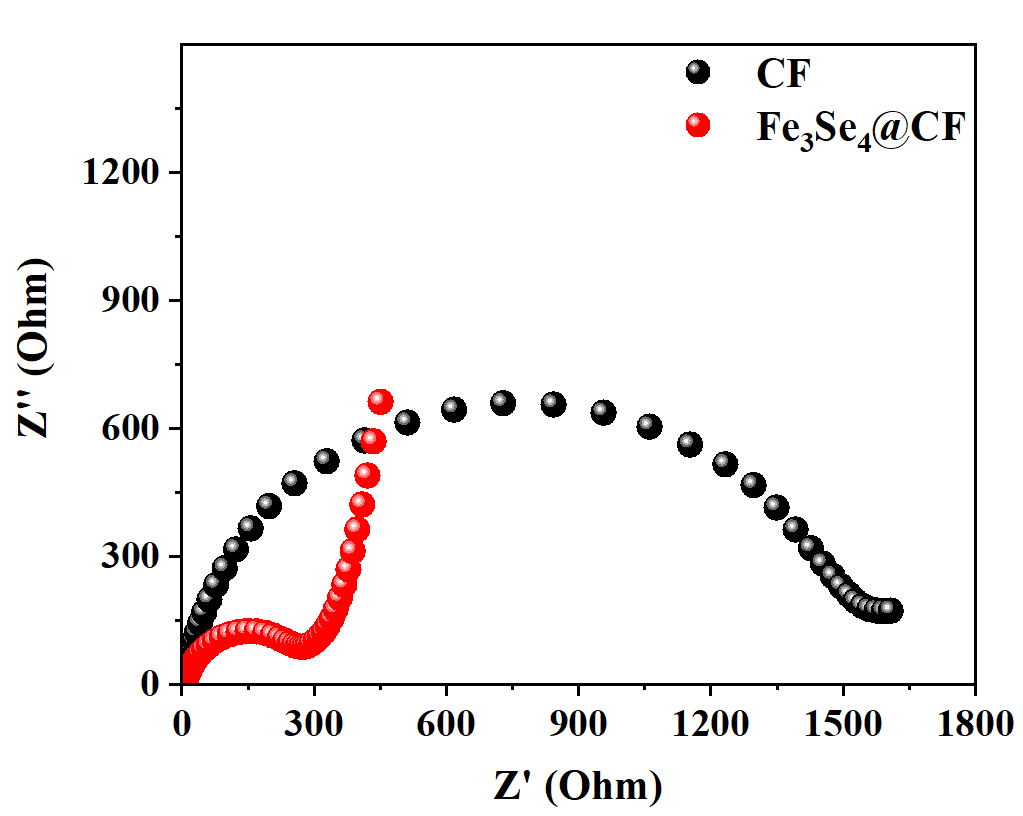


**Figure S6.** KIB analysis. EIS analysis of CFs and Fe_3_Se_4_@CFs
